# Facilitators Associated with Nursing Burnout in the Ambulatory Care Setting as COVID-19 Subsides: A Rapid Review

**DOI:** 10.3390/healthcare11152122

**Published:** 2023-07-25

**Authors:** Cristian Lieneck, Jolene Bair, Stephanie Ardell, Brittany Aldridge, B. J. Austin

**Affiliations:** 1 School of Health Administration, Texas State University, San Marcos, TX 78666, USA; 2 School of Health Sciences, Southern Illinois University-Carbondale, Carbondale, IL 62901, USA; jolene.bair@siu.edu (J.B.); stephanie.nesmith@siu.edu (S.A.); brittany.love@siu.edu (B.A.); bradley.austin@siu.edu (B.J.A.)

**Keywords:** nursing, burnout, workforce, COVID-19, coronavirus, ambulatory care, outpatient

## Abstract

The COVID-19 pandemic has significantly impacted the healthcare industry and its workforce, particularly nurses, who have been at the forefront of patient care. As the world begins to emerge from the pandemic, attention is turning to the long-term effects of the crisis on nurses’ mental health and well-being, and specifically nursing burnout. Prevalent risk factors related to nursing burnout often historically involve high workload, insufficient support and/or resources, work–life imbalance, and even lack of autonomy and organization climate challenges. Understanding the factors that contribute to nursing burnout to help mitigate it is vital to ensuring the ongoing health and well-being of the nursing workforce, especially since the ongoing waning of coronavirus (COVID-19). This rapid review identifies 36 articles and explores the latest research on nursing burnout in outpatient (ambulatory care) healthcare facilities as the global pandemic continues to subside, and therefore identifies constructs that suggest areas for future research beyond previously identified contributing factors of nursing burnout while the pandemic virus levels were high.

## 1. Introduction

### 1.1. Introduction to the Problem

The COVID-19 pandemic of 2020 flipped the world of healthcare as we know it on its head. It took an already overworked, understaffed industry and added even more pressure with waning resources, absent administration, poor work–life balance, and an even bigger increase in the already high turnover rates. Nurse burnout refers to a state of physical, emotional, and mental exhaustion that results from prolonged exposure to high levels of job-related stress, demanding work conditions, and a sense of depersonalization or cynicism towards one’s job. It is a complex and multi-dimensional phenomenon that can occur when nurses experience overwhelming feelings of being emotionally drained, depleted of energy, and unable to cope effectively with the demands of their work.

Prior to the pandemic, nurses were more willing to let the long-established problems with the healthcare industry slide, but with the pandemic exacerbating these issues, coupled with the extreme compassion fatigue and burnout that are being experienced, they are no longer staying quiet and sweeping them under the rug. This has led to nurses leaving the bedside and the industry in droves, increasing the extreme nursing shortage even more. Burnout can have serious consequences for both the well-being of the individual nurse and the quality of patient care provided. It can lead to increased absenteeism, higher turnover rates, decreased job satisfaction, and compromised patient safety and outcomes. Recognizing and addressing nurse burnout and related impacting risk and protective factors is crucial for promoting the well-being of nurses and ensuring the delivery of high-quality healthcare services. Implementing supportive strategies, providing resources, and creating a positive work environment can help mitigate the risk of nurse burnout and promote a healthier and more resilient nursing workforce.

### 1.2. Significance of This Study

Prior to 2022, the COVID-19 pandemic progressed in waves across countries as they worked to adapt to public health measures and developing treatment protocols. Throughout this whole period, professionals in the nursing field experienced continued burnout as a result of the pandemic. Multiple systematic literature reviews were conducted as COVID-19 surged across the globe and identified contributing factors, to include the following constructs:

- Emotional and physical exhaustion [1,2,3];
- Responsiveness of organizational reaction to virus surges [2];
- Lack of identification methods of burnout among nursing teams during the provision of care [1];
- Working environment, strength of staff members, and leadership effectiveness [3].

These prior review findings were from studies conducted in acute care (hospital) settings, the primary location for the treatment of COVID-19 patients during the initial and ongoing stages of the pandemic. In addition to identifying nursing burnout constructs, these prior reviews further identified and discussed potential long-term industry workforce implications, including quality of care and patient outcomes [4]. Now that the COVID-19 pandemic has subsided, with increasing vaccination rates and a return to routine care for healthcare facilities, our research team was interested in current nursing burnout contributors specific to outpatient (ambulatory care) healthcare organizations. Therefore, the aim of this study is to better identify the current and most recent nursing burnout factors in the outpatient/ambulatory care segment of the healthcare industry. Such information could support healthcare leadership’s efforts to combat ongoing burnout factors in the nursing workforce, while also staying attuned to underlying factors contributing to the issue in the routine care (non-acute) environment. While our review team recognizes that the decline of the COVID-19 virus and its virulence will vary across both time periods and locations (region), the intent of this study is to attempt to preempt any new or developing influences upon the ongoing burnout experienced in the industry’s nursing profession.

## 2. Materials and Methods

This systematic review was guided by the Preferred Items for Systematic Reviews and Meta-Analysis (PRISMA) protocol. The 2020 PRISMA checklist and flow diagram were used in an effort to demonstrate process transparency and the thoroughness of the research team’s rapid review process and related research databases.

### 2.1. Overview

The authors of this manuscript constituted the entire research team for this study. Our research team’s overall intent was to investigate underlying factors (constructs) influencing burnout in the field of nursing for ambulatory care (outpatient) healthcare organizations in the present day. Three main research databases were queried using the EBSCOhost platform via the Southern Illinois University-Carbondale library website: Academic Search Complete, CINAHL Plus, and Health Source: Nursing/Academic Edition.

### 2.2. Inclusion Criteria

Our research team used multiple iterations of review search terms to identify a search string that encompassed as many articles related to the review topic as possible. Proper identification of current publications (article manuscripts) surrounding the ongoing nursing shortage in the healthcare industry began with basic search engine (Google, etc.) searches to identify initial terminology related to the study topic. Next, Medical Subject Headings (MeSH), the controlled vocabulary thesaurus that indexes publications for PubMed, was also searched. Finally, the library’s EBSCOhost advanced search preempted (suggested) terminology was also utilized.

Multiple iterations of searches were conducted with varying Boolean operators to identify the largest possible review sample for this study. The final search string identified by the team was as follows:

[(“nursing shortage’) AND (“ambulatory care” OR “outpatient care” OR “outpatient services” OR “urgent care” OR “clinic visits”) AND (“burnout OR burn-out OR burn out OR stress OR occupational stress or compassionate fatigue”)]

After an initial search, which yielded 6859 articles, our research team narrowed down the results by applying filters for publication dates between 1 January 2022 and 2024, leading to the identification of 343 articles. The selection of the 1 January 2022 date was crucial as it allowed the team to focus on understanding the reasons behind nursing-related workforce burnout in the ambulatory care setting, considering the decreasing rates of COVID-19 transmission and the alleviating concerns of the pandemic within the industry. While the incidence and prevalence of the virus varied across countries at any one time, the 1 January 2022 to present publication date search criteria were selected by our research team in an attempt to identify potential constructs for the research topic during the initial stages of increasing vaccination rates and a significant drop in the spread of the virus during this timeframe.

### 2.3. Exclusion Criteria

Published manuscripts were included in the review if nursing shortages were specifically addressed, as related to burnout challenges and/or underlying themes. Publications had to be published in good-quality, peer-reviewed journals and/or identified as academic journals by the EBSCOhost database library website. Our research team implemented supplementary database search parameters to generate targeted and relevant results that aligned with the specific research objectives. In addition to the 1 January 2022 through 2024 publication date criteria (−6516 articles), full-text filtering was also applied (−39 articles). Peer-reviewed articles only were filtered again using the EBSCOhost database option, resulting in 162 articles remaining (−142 articles). The option to further delineate down to academic journals only and English-only articles did not result in the removal of any further manuscripts beyond this point (−0 articles).

**Figure 1:**
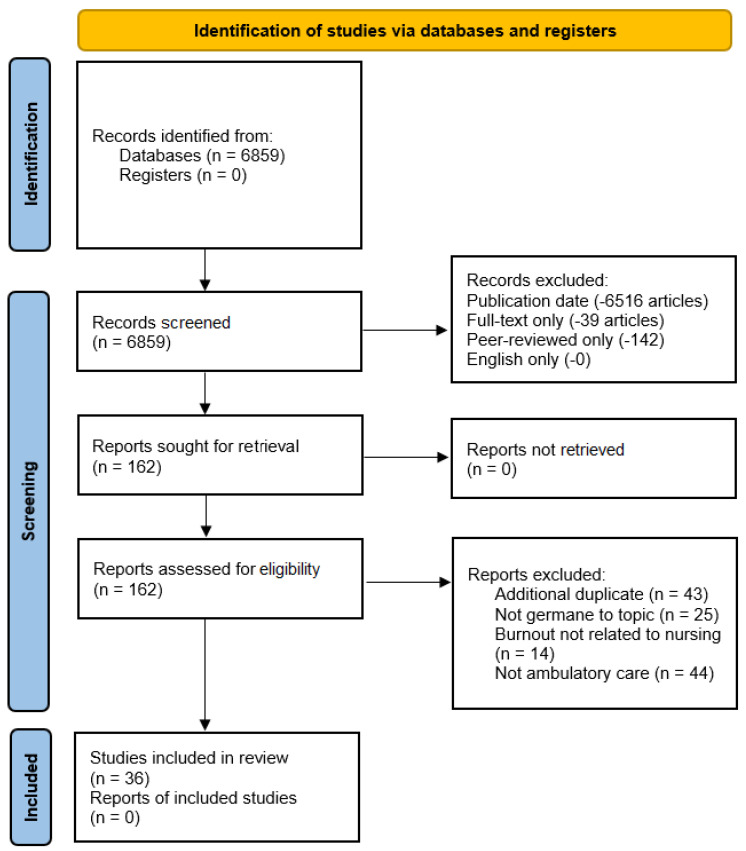
Preferred Reporting Items for Systematic Reviews and Meta-analysis (PRISMA) figure that demonstrates the study selection process.

Using the available menu/check-box filter options on the EBSCOhost platform, our research team carried out all the exclusion steps mentioned above. Figure 1 visually represents the rapid review process and the search exclusion criteria applied, leading to a final set of 162 articles.

The authors of this study conducted a comprehensive review of the included studies, involving a thorough examination of each identified publication by at least two members of the research team. Table 1 presents the distribution of three sets, each containing 162 review articles, which were assigned to individual research team members.

Efforts included in the full-text review of the remaining manuscripts included the assessment of each manuscript’s patient population, intervention, comparison, and outcome(s) (PICO) variables, including assessing each article’s research method(s), validity, and the reliability of data. This assessment by our research team occurred only after the application of the EBSCOhost search parameters. After conducting a full-text review for eligibility, our research team identified 36 publications that met the criteria and were retained for further analysis. The articles removed from the review to result in the remaining 36 were excluded for reasons as categorized below:

- In total, 43 articles were identified as additional duplicates not identified previously by the research database.
- Out of the total, 25 articles were found to be irrelevant to the research topic. These articles were either mistakenly included in the initial database search or mentioned “nursing” in the context of healthcare delivery but were not directly related to “nursing burnout” or similar subjects. Of these, 14 articles were specifically focused on burnout in the health professions, but not specific to the field of nursing (i.e., physicians, therapists, other medical providers).
- In total, 44 articles were removed for focusing on themes related to this study but not specific to the outpatient/ambulatory care setting.

To address potential article bias or conflicts related to the application of selection criteria, our research team engaged in online collaboration through webinars. During multiple consensus meetings, no disagreements arose among the team members on these matters.

## 3. Results

### 3.1. Study Characteristics

The team’s full-text review of the 36 articles identified underlying constructs (characteristics) associated with nursing burnout in the ambulatory care/outpatient healthcare settings, specifically, from the database search date filter, those published from 1 January 2022 through 2024. A summary of the review findings for each article is provided in Table 2.

### 3.2. Identification of Underlying Constructs

**Figure 2:**
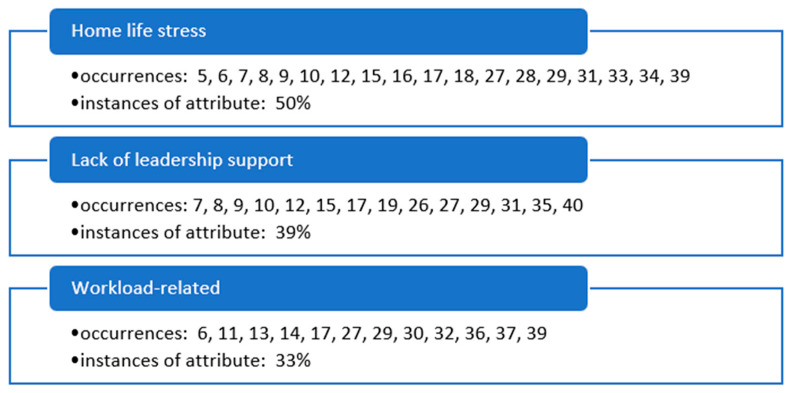
Primary occurrences of underlying themes (constructs) influencing nursing burnout in ambulatory care organizations as identified in the literature.

Early in the review team’s consensus meetings, three primary themes (underlying constructs) were identified in the literature, supporting the research topic of facilitators associated with nursing burnout in the ambulatory care setting as COVID-19 subsides (Figure 2).

Home life stress was a factor of nursing burnout during this timeframe of the pandemic in ambulatory care organizations at 50% instances of attribute contribution. This identified construct was observed the most in the literature of all the identified constructs and was a significant issue, as discussed in the identified articles. A lack of outpatient facilities, leadership support, and workload-related issues were also identified by the research team as significant contributing factors, at 39% and 33% instances of attribute each in the review, respectively.

## 4. Discussion

Each identified construct in this review supports the research team’s initial hypothesis that significant contributors to burnout in the field of nursing are continuing as the global pandemic continues to subside. With higher vaccination rates, lower transmission of the virus, and even fewer acute symptoms related to infection, the burnout of nurses in the healthcare industry continues, including in outpatient care facilities. As prior research identified contributing factors specifically related to the COVID-19 virus and related nursing personnel threats [1,2,3], this review identified what can be considered more routine or non-pandemic-related contributing factors to nursing burnout as COVID-19 continues to subside.

### 4.1. Home and/or Work Stress Contributing to Nursing Career Burnout

Health professionals in the nursing field are struggling regardless of specialty area. Nurses are leaving their current positions and, in some cases, the healthcare field entirely, seeking careers that are significantly less stressful and safer to work in [6,16,39]. Research has shown that career burnout is associated with both home and work stress. Work–family conflict is work and family roles being incompatible, causing tension in the home and at work [5,16,29]. This incompatibility has majorly contributed to the extreme nursing shortage we are experiencing in today’s health industry. It will be crucial to the future success of the healthcare industry to evaluate these stressors and address them accordingly.

About one third of an individual’s life is spent at the workplace, which can be a strain on one’s mental health and impede the ability to provide quality care for patients as well as providing for their loved ones at home [5,16,33,39]. Contributors of work stress include unsafe working conditions, increased working hours, and increased patient loads. The COVID-19 pandemic brought about a strenuous working environment for nurses, causing an increased feeling of burnout [8,10]. Personal protective equipment was limited, and large influxes of patients at one time resulted in longer hours and unsafe nurse-to-patient ratios [22,39]. Nearly 3 nurses per 1000 were abandoning their positions at bedside, leaving the nurses that were left to take on each of these dilemmas [5,10,12]. Solutions to addressing these issues were identified in various studies and included enhancing interprofessional teamwork, providing improved occupational health services, adjusting working hours, and providing continuous mental health services to employees [6,8,15]. Each of these areas of improvement have been found to curb the intentions of nurses to quit.

Burnout affects nearly half of all nurses, and these numbers are even higher in certain specialties [6,8,15]. Though work–life has a direct correlation to burnout, studies have also found associations of home life and sociodemographic factors to be contributors to the high prevalence of nurse burnout [15]. In one study, nurses were surveyed to evaluate the correlation between job burnout, professional quality of life, and home conflicts and the interference with work. The three-part questionnaire found that factors such as having children, living with parents, having a disabled family member at home, and having a second job were all significant factors that contributed to professional quality of life [5]. Studies to address these concerns suggest that customizing emotional and mental programs to respond to high-risk profiles will be beneficial to both employees and the organization as a whole [6,8,9,15]. The success of an organization and avoidance of employee burnout truly rely on an organization’s ability to realize and address the aforementioned factors and help nurses establish a positive work–life balance in order to attract new employees and retain the staff they currently have.

### 4.2. Lack of Organization/Administration Support

Recent studies have shown that burnout has been linked to nurses’ intention to quit the profession [8,26,35]. It was reported that moderate to high burnout has been reported among nurses and other healthcare professionals [7,8,26]. High workload, years of practice, resilience, inadequate staffing, and lack of leadership support have been identified as some of the highest complaints amongst healthcare workers. In one study, a prevalence rate of 69.0% of nurses with intention to quit their profession was reported [7].

According to Poku, Donkor, and Naab, another study carried out in Ghana reported health labor force scarcities have consequences on global healthcare delivery and quality of patient care [10]. Understanding the challenges of turnover rate and the retention of staff is essential in the discussion of strategies for improving the nursing workforce. High staff turnover intentions in many organizations are attributed to factors such as poor quality of staffing and inadequacy of proper equipment to care for patients. Such poor work conditions present high work demand, inadequate group support, and increased physical and emotional work demand. Lack of support from nurse managers, unjustified workloads, and increased emotional exhaustion of RNs mostly lead to increased staff intentions of resigning [10]. 

A study conducted in Oman by Sabei et al. found that nursing turnover may adversely affect the delivery of healthcare services and potentially threaten patient safety outcomes [15]. Nursing turnover has been associated with increased healthcare costs and additional financial burdens for organizations. The COVID-19 pandemic has further exacerbated this issue, with evidence showing that an increasing number of nursing professionals have left the bedside and intend to leave their jobs due to the threat posed by the virus. Therefore, the World Health Organization and many healthcare policymakers have emphasized the urgent need to invest in nurses to strengthen global health. Identifying the factors influencing nurses’ intentions to leave is a crucial step in enhancing retention and improving overall patient safety [7,8,15,30,32,39].

### 4.3. Workload-Related Issues

The COVID-19 outbreak caused a serious increase in workload in the healthcare field. Due to the increasing number of patients during the outbreak, there were not enough workers to cover the long shifts that needed to be covered. Many nurses had a lot of struggles to overcome with the long hours of work. There was an increase in nurses becoming burnt out in their profession and quitting their jobs, only to cause harder times and workloads for other nurses. Many nurses complained of conflicts with home life and family time. They could not spend the time they needed to with their families due to working schedules. These long hours caused even more nurses to quit their jobs and also contributed to a greater number of dysfunctional families.

The overwhelming workload was caused by increased patients and a decrease in workers. The same problems are being experienced worldwide. There have been many days where there was a 50–60% increase in the daily workload [11,37]. Due to the complexity of healthcare, when there is one portion of the business that is being overworked or understaffed, it affects multiple areas of the industry, not just nursing. Nurses already work long hours and when they are required to work extra it causes a domino effect. Fully staffing one department by utilizing the staff of another leads the helping unit potentially being understaffed. Then, trying to staff that unit leads to another unit being short-staffed, and so on. Moving staff around between units in a hospital is not a sustainable solution, and has only contributed more to nurses becoming frustrated with their jobs [13,27,29]. 

When the process of being overworked continues, many problems arise. In one study, 300 nurses who reported experiencing high levels of workplace burnout also had signs of depression. They were also not living a healthy lifestyle and starting to show weight gain. These same nurses agreed that they were not able to complete all management duties efficiently and effectively due to workload. None of them were happy with the way their lives have been through the stress of COVID-19 and all of the extra work it was taking to keep things up and running in outpatient medical facilities [6,8,17,29].

### 4.4. Ambulatory Care Nursing Burnout as the Pandemic Subsides

This rapid review identified underlying themes related to nursing burnout in the outpatient/ambulatory care healthcare industry, which can easily be mapped to pre- and mid-pandemic challenges [1,2,3] as well. While these underlying variables identified by the research team do not add any additional or new influencing constructs to the ongoing industry challenge experienced by healthcare organizations and their leaders, the findings do support a continued requirement to address this important industry dilemma. While balancing home life with workplace expectations and needs, a lack of organizational leadership support, and other work-related variables were identified in this review as contributing factors to nursing burnout in the ambulatory care setting, healthcare organizational leadership should continue to assess and address the need to reverse such contributing factors as COVID-19 continues to subside and outpatient care, specifically any postponed or delayed routine care, resumes in this industry segment.

## 5. Conclusions

For a long time, nurses have experienced compassion fatigue and burnout in the healthcare industry. Improper work–life balance, lack of administrative support, and being overworked and underappreciated are the biggest contributing factors to these feelings. A lack of response to these issues, especially with the added pressure of the COVID-19 pandemic, has led to nurses leaving the bedside and the profession in droves. As demonstrated in this review, current contributing factors leading to nursing burnout have changed minimally since the peak of the global pandemic [1,2,3] and the underlying contributing factors continue to exist.

This rapid review has multiple limitations, as with any qualitative study. The review only assesses nursing burnout in the ambulatory care segment of the healthcare industry, which does vary across multiple (national) healthcare systems. Characteristics specific to individual healthcare systems will most likely have inherent influencing variables related to nurse burnout, and therefore should be investigated as future research. The review includes a limited publication (manuscript) date range to determine if any new underlying constructs related to nursing burnout in ambulatory care exist, as compared to previously known influencing variables. This date range search parameter is not perfectly assignable to the decline in COVID-19 prevalence across all regions and levels of virulence. As a result, the findings of this review should be interpreted generally, yet do support previously known factors contributing to nursing burnout in the healthcare industry.

In order to remedy the extreme nursing shortage we are experiencing, future research and leadership initiatives should address the root causes of nurses experiencing burnout as the pandemic continues to subside. Actions to support such initiatives may include listening to employees, supporting employees in establishing a healthy work–life balance, and implementing solutions to satisfy and retain their employees for the long term in outpatient care organizations.

## Figures and Tables

**Table 1:** Reviewer assignment of the initial database search findings (full-article review).

Article Assignment	Reviewer 1	Reviewer 2	Reviewer 3	Reviewer 4	Reviewer 5
Articles 1–40	X	X	X		
Articles 41–80	X	X	X		
Articles 81–120	X			X	X
Articles 121–162	X			X	X

**Table 2:** Summary of findings (*n* = 36).

Reference Number and Authors(s)/Year	Article Title	Journal/Publication	Participant Population	Purpose/Method	Outcome/Observation(s)
[5] Dilmaghani et al., 2022	Work-family conflict and the professional quality of life and their sociodemographic characteristics among nurses: A cross-sectional study in Tehran, Iran	*BMC Nursing*	Nurses in Tehran, Iran	Cross-sectional Correlation of job burnout and professional quality of life was due to home conflicts that interfere with work.	Professional/quality of life factors identified. Home life/conflict significantly affected job burnout for various reasons. Other reasons for nursing burnout included disabled family members at home, child less than 1 year of age, living with parents, having a second job, and single individuals.
[6] Girard and Nardone, 2022	The Oregon Wellness Program: Serving Healthcare Professionals in Distress from Burnout and COVID-19	*Journal of Medical Regulation*	Healthcare professionals involved with the Oregon Wellness Program	Descriptive/program Analysis of the effectiveness of the Oregon Wellness Program initiative in assisting healthcare workers overcome burnout in relation to COVID-19 was conducted. Program satisfaction surveys were distributed to those who utilized the mental health services to evaluate their effectiveness.	Surveys indicated satisfaction with the program, showing positive outcomes in individuals’ personal lives. Recommended changes were implemented, and positive change was seen. Burnout rate decreased with an increase in program utilization.
[7] Opoku et al., 2022	Attrition of Nursing Professionals in Ghana: An Effect of Burnout on Intention to Quit	*Nursing Research & Practice*	Nursing professionals in Ghana	This study evaluated the effect of burnout on intention to quit the nursing profession. Cross-sectional study completed with 300+ participants using the Maslach Burnout Inventory survey, which determines the association between burnout and potential possibility of quitting.	Nurses’ intentions to quit their current positions were assessed. Nearly half of the participants were experiencing depression, emotional exhaustion, and low personal accomplishment. The same number were also considering quitting the profession altogether. The findings suggest that it is imperative to identify resources to assist nurses in not reaching the point of exhaustion and depression so as to prevent burnout and losing employees.
[8] Udoh and Kabunga, 2022	Burnout and Associated Factors among Hospital-Based Nurses in Northern Uganda: A Cross-Sectional Survey	*BioMed Research International*	Hospital-based nurses in N. Uganda	The study aimed to assess the burden of burnout and associated factors among nurses in Northern Uganda. Self-administered questionnaires were issued to over 300 nurses in Uganda and descriptive statistics were assessed.	Most participants were female and high levels of burnout were prevalent. Factors associated with burnout included age, social support, healthy eating, workload, and management responsibilities. Suggestions to reduce burnout experiences included recruiting additional nurses to decrease workload and/or adjusting working hours to decrease workplace-related burnout.
[9] Thapa et al., 2022	Job demands, job resources, and health outcomes among nursing professionals in private and public healthcare sectors in Sweden—a prospective study	*BMC Nursing*	Nursing professionals in Sweden	Prospective study Evaluated nursing job demands and resources in relation to health outcomes by comparing between the private and public health sectors. Over 1000 nurses and nurse assistants were surveyed over the span of 2 years. The Swedish Longitudinal Occupational Survey of Health was used to collect data.	Health outcomes were assessed and related to nursing burnout factors. Resources in the public healthcare system were found to be deficient as low social support, low control, and higher threat were discovered as compared to the private sector. Strengthening job resources in the private and public sectors to assist with work-related health was recommended to help alleviate nurse burnout.
[10] Poku et al., 2022	Impacts of Nursing Work Environment on Turnover Intentions: The Mediating Role of Burnout in Ghana	*Nursing Research & Practice*	Nursing professionals in Ghana	Descriptive study The study worked to determine impacts of work environment on burnout and turnover intentions of RNs in Ghana. Over 200 nurses were surveyed with questions regarding perceptions of the work environment, burnout, and turnover intentions.	Nursing turnover intentions were identified. Many nurses had a positive outlook on their work environment; however, a greater number had quitting intentions. Unsafe work environments impact nurse retention and significantly impacted their responses in the study.
[11] Litke et al., 2022	Building resilience in German primary care practices: a qualitative study	*BMC Primary Care*	Primary care practices in Germany	Qualitative study design. The study explored what situations are perceived as a crisis by primary care organizations and identified strategies to overcome issues related to provider/team burnout. Focus groups were used to collect data from groups of different personnel (including nurses) within a typical primary care practice.	Multiple team/stakeholder perceptions were identified as crises. Many issues were internal to the facility and even health-system-wide management-related issues. The findings suggest that resilience among employees within an ambulatory care organization exists to help prepare and even overcome (or prevent) personnel crises.
[12] Yosef et al., 2022	Occupational Stress among Operation Room Clinicians at Ethiopian University Hospitals	*Journal of Environmental and Public Health*	Operation room clinicians at Ethiopian university hospitals	Descriptive study analysis. The study evaluated the prevalence of and factors associated with workplace stress among operation room clinicians at healthcare facilities in Ethiopia. Almost 400 clinicians were surveyed using the United Kingdom Health and Safety Executive’s Management Standards Work-related Stress Indicator Tool to assess workplace stress.	The findings demonstrate a prevalence of workplace stress at 78.4% among survey respondents. Factors contributing to stress/burnout in the study included rotating shifts, work weeks of over 80 h, being a nurse within the facility, and being an anesthetist within the facility. An additional recommendation included providing improved occupational health services.
[13] Bodenheimer, T., 2022	Revitalizing Primary Care, Part 2: Hopes for the Future	*Annals of Family Medicine*	n/a	Survey panels were created to evaluate the effectiveness of improvement initiatives related to outpatient practice burnout.	The findings suggest that root causes of primary care problems are attributed to a low percent of expenditure dedicated to primary care and also unmanageable large patient loads, resulting in burnout and poor patient access. Suggestions include the establishment of practices that initiate strategies that are sustainable. However, additional funds are needed to create powerful teams to implement these.
[14] Sirkin et al., 2023	Primary Care’s Challenges and Responses in the Face of the COVID-19 Pandemic: Insights from AHRQ’s Learning Community	*Annals of Family Medicine*	Primary care practitioners and nursing professionals during COVID-19 pandemic	Descriptive study. AHRQ-initiated learning community that connected clinicians to resources that specifically assist, support, and grow primary care practices as a result of pandemic-related challenges (including burnout).	Five domains of challenges and responses emerged from the learning community. The engagement of the community identifying real-time solutions to challenges during the pandemic allowed for valuable insight for future research and policy.
[15] Al Sabei et al., 2022	Relationship between interprofessional teamwork and nurses’ intent to leave work: The mediating role of job satisfaction and burnout	*Nursing Forum*	Inter-professional healthcare teams	Cross-sectional study. The study evaluated the direct and indirect effects of interpersonal teamwork on nurse intentions to leave due to job satisfaction and burnout. A questionnaire was distributed to over 2000 nurses that measured teamwork, job satisfaction, burnout, and potential intention to leave. The direct effect of teamwork was specifically measured.	Inter-professional teamwork was assessed and the intent to leave work, mediated by job satisfaction, was observed. Teamwork at work was found to be directly associated with nurses’ intentions to leave. Job satisfaction and job burnout were indirectly associated with nurses’ intention to quit. Recommendations include enhancing interprofessional teamwork to assist in reducing nurses’ intentions to leave their profession and ultimately reduce nursing shortage.
[16] Terp et al., 2022	A feasibility study of a cognitive behavioral based stress management intervention for nursing students: results, challenges, and implications for research and practice	*BMC Nursing*	Nursing students	Feasibility study to assess cognitive-behavioral-based stress management intervention. An assessment of the feasibility and acceptability of a newly developed cognitive behavioral stress management intervention for nursing students was conducted. Standardized measurements and questionnaires were created to measure recruitment capability, intervention acceptability, and preliminary evaluation of participant psychological changes.	Feasibility was found for potential intervention acceptability, data collection procedures, and adherence. Challenges were identified within the recruitment capability for nurses. The intervention was found to be feasible and beneficial to nursing students and related feelings of burnout.
[17] Poon et al., 2022	A global overview of healthcare workers’ turnover intention amid COVID-19 pandemic: a systematic review with future directions	*BMC*	Healthcare workers	The study examined factors affecting turnover intention among healthcare workers during COVID-19. Several databases were searched for systematic reviews for insight into the issue.	The turnover intention of healthcare workers amid the pandemic was identified. The multitude of studies revealed five themes: fear of COVID-19 exposure, psychological responses to stress, socio-demographic characteristics, adverse working conditions, and organizational support. Employee turnover was found to be influenced by many factors; however, working conditions, burnout, and specific vulnerable groups should be of top priority in such emergent times.
[18] Park et al., 2022	Latent profile analysis on Korean nurses: Emotional labor strategies and well-being	*Journal of Advanced Nursing*	Korean nurses	Latent profile analysis was conducted. The study examined the emotional labor management strategies required in the workplace among Korean nurses. Deep acting and genuinely experiencing emotions required in the workplace was also assessed. Latent profile analysis was distributed to over 200 nurses working in university hospitals for the study.	The results indicate that those with high levels of surface acting also had high levels of emotional exhaustion and turnover intentions. Customizing emotional labor management policies to respond to high-risk profiles will be beneficial to help alleviate nurse burnout in the workplace.
[19] Anskär at al., 2022	‘But there are so many referrals which are totally … only generating work and irritation’: a qualitative study of physicians’ and nurses’ experiences of work tasks in primary care in Sweden	*Scandinavian Journal of Primary Health Care*	Physicians and nurses in primary care	Qualitative study design. Exploration of the perceptions of physicians and nurses in the primary care setting was conducted to assess the meaningfulness of their work and the use of their professional competence. Interviews with nurses and physicians were conducted at publicly managed primary care centers that included a wide range of large and small practices to gather the most accurate data.	Many clinicians reported that their individual competencies were appropriately used. Several responses indicated work-related difficulties due to lack of resources, challenging electronic data systems, and work inefficiencies. Illegitimate or inappropriate work tasks may result in discontentment and lead to negative work stress, causing burnout.
[20] Shen et al., 2022	Analysis of the Effect of the Communication Ability of Nurses in Outpatient Infusion Room on the Treatment Experience of Patients and Their Families	*Computational & Mathematical Methods in Medicine*	Nurses in outpatient infusion rooms	This descriptive study attempted to observe the effect of nursing communication on ability in an outpatient infusion room and its effect on the treatment experience of patients and their families. The study analyzed whether improving nurses’ communication skills can reduce the incidence of doctor–patient disputes.	By improving communication skills for nurses, quality management can improve the mental toughness and hope level of patients, which is conducive to controlling their emotions and avoiding the occurrence of doctor–patient disputes.
[21] Dewey and Allwood, 2022	When Needs Are High but Resources Are Low: A Study of Burnout and Secondary Traumatic Stress Symptoms Among Nurses and Nursing Students in Rural Uganda	*International Journal of Stress Management*	Nurses and nursing students in rural Uganda	A cross sectional study was conducted with a sample size of 208 healthcare workers (RNs, nursing students), self-reported questionnaires were completed, and data were analyzed in areas such as trauma load, symptoms of burnout, PTSD, and secondary traumatic stress. The study aimed to evaluate healthcare workers, especially those working in low-resource settings, and how they may be at an increased risk for work-related stress problems such as burnout and secondary traumatic stress. Also explored the association between intent to leave the profession and nursing shortage in Uganda.	Burnout and secondary traumatic stress symptoms among nurses and students identified in the study. The results indicate that exhaustion and burnout lead to increased trauma loads, which ultimately leads to symptoms of secondary traumatic stress and PTSD.
[22] Poku et al., 2022	Quality of work-life and turnover intentions among the Ghanaian nursing workforce: A multicenter study	*PloS one*	Ghanaian nursing workforce	Descriptive study evaluating the quality of work–life in regard to the nursing workforce and nursing turnover in Ghana. Data were collected using the Work-Related Quality of Life Scale and the Turnover Intention Scale. Over 300 registered nurses in the primary, secondary, and tertiary care settings were sampled.	Quality of work–life and turnover intentions among Ghanaian nurses identified. The results indicate that nurses perceive quality as low, with nearly half of the nurses having intentions to quit. Predictors of turnover intentions include number of years in the healthcare industry, general well-being, job control and satisfaction, and working conditions.
[23] Tolksdorf et al., 2022	Correlates of turnover intention among nursing staff in the COVID-19 pandemic: a systematic review	*BMC Nursing*	Nursing staff during COVID-19 pandemic	Study evaluating factors associated with nurse turnover during COVID-19 to assist in future effort to prevent nursing shortage in general and during a pandemic.	Correlates of turnover intention among nursing staff in COVID-19 pandemic include health factors, psychological factors, and demographic characteristics—all were found to be associated with nursing turnover and retention. Organizational factors that are associated with high turnover include caring for severely ill patients, high job demands, and moral distress surrounding COVID-19. Resilience and supporting leadership could mitigate adverse associations and decrease turnover intentions.
[24] Hegarty et al., 2022	Nurse staffing levels within acute care: results of a national day of care survey	*BMC Health Services Research*	Acute care nursing staff in the UK	Descriptive study. The study evaluated the relationship between nurse staffing levels and patient safety and also sought to identify whether inadequate nursing staff is associated with increased medical error as well as higher morbidity and mortality. In total, 122 acute hospitals were surveyed using the Society for Acute Medicine Benchmarking Audit.	The results demonstrate that 81.9% of units achieved the daytime benchmarks (1:3.4) and 60.6% of units achieved the nighttime benchmarks (1:6). A deficit of 4128 h was identified in the time it takes to perform nursing duties compared with staffing ratios. The study concludes that there is a nursing shortage within acute care as staffing ratios at many facilities are below benchmark.
[25] Paulus, 2022	Exploring the Evidence: Considerations for the Dialysis Practice Setting Approach to Staffing	*Nephrology Nursing Journal*	Dialysis practice setting	Descriptive research providing evidence on approaches to staffing shortages in the dialysis sector/private practice setting by defining core elements when designing a staffing model. Various databases were evaluated, and questionnaires submitted by readers of the *Nephrology Nursing Journal* were analyzed.	Considerations for the dialysis practice setting approach to staffing required to address turnover issues. Development of a healthy work environment that prioritizes patients over profit and ultimately supports patient care, achieving high-quality outcomes, and assists with staffing challenges.
[26] Kühl et al., 2022	General practitioner care in nursing homes during the first wave of the COVID-19 pandemic in Germany: a retrospective survey among nursing home managers	*BMC Primary Care*	Nursing home managers in Germany	Retrospective survey design. Study exploring the detrimental impact of COVID-19 on practitioner care in nursing homes. A retrospective online survey was conducted, with responders being nursing home managers during the first wave of COVID-19.	It was reported that nursing homes had no deficits in routine visits or acute visits by general practitioners. Analysis revealed that deficits that were prevalent regarding routine visits were associated with visiting restrictions for general practitioners and nursing home size. New concepts for general practitioner care should be implemented for emergent response plans to ensure consistent and reliable care.
[27] Midje et al., 2022	The role of working environment and employee engagement in person-centered processes for older adults in long-term care services	*International Practice Development Journal*	Employees in long-term care services	A cross-sectional survey design and descriptive analysis with standardized self-report questionnaires were used to collect data on job resources, job demands, work engagement, and person-centered processes. In total, 128 registered nurses and nursing assistants were included in the sample.	Job demands and job resources are distinct aspects of the working environment that interact in predicting staff well-being and motivation. Work engagement can serve as a means to improve job motivation and performance potentially helps facilitate activities that operationalize person-centered practice. Work engagement and person-centered processes were positively associated with job resources.
[28] Heuel et al., 2022	Chronic stress, behavioral tendencies, and determinants of health behaviors in nurses: a mixed-methods approach	*BMC Public Health*	Nurses	The study explored barriers and resources associated with health behaviors in nurses with different stress levels and work-related behavioral tendencies. Health behavior determinants were identified by the researchers. A mixed-methods transformative triangulation design was utilized for a sample of nurses (*n* = 43) who filled out chronic stress and work-related behavior and experience patterns questionnaires. Semi-structured interviews were also conducted.	Chronic stress, behavioral tendencies, and determinants of health behaviors were identified and associated with nursing burnout. The results indicate that 84% percent of the nurses were chronically stressed, while 49% exhibited unhealthy behavioral tendencies in the workplace.
[29] Jingxia et al., 2022	The changes in the nursing practice environment brought by COVID-19 and improvement recommendations from the nurses’ perspective: a cross-sectional study	*BioMed Central*	Nurses during COVID-19 pandemic	A cross-sectional study was conducted among 460 nurses from seven hospitals in Sichuan, China. Quantitative and qualitative data were collected from an online questionnaire.	Changes in nursing practice environment and improvement recommendations during the pandemic help address nurse turnover issues. The findings indicate the score of the total scale and the dimensions were significantly higher than the norms.
[30] Phiri et al., 2022	International recruitment of mental health nurses to the national health service: a challenge for the UK	*BMC Nursing*	Mental health nurses recruited to UK NHS	Descriptive study addressing the importance of concentrating on recruiting new staff and retaining current staff during the COVID-19 pandemic.	A primary strategy for the resolution of nursing shortages at facilities is the recruitment of internationally trained mental health nurses.
[31] Jithitikulchai, 2022	Improving allocative efficiency from network consolidation: a solution for the health workforce shortage	*BMC*	n/a	Conceptual study design comparing the workload per worker and economic valuation of area-based networks or ex ante scenarios with the hospital-level or status quo scenario.	Network consolidations of primary-level hospitals within the same district could reduce workload per worker by seven percentage points. Another practical policy option is to consolidate similar hospital levels such as primary, first-level secondary, and mid-level secondary hospitals all together within the same province, which could result in the reduction in the workload per worker by 6–7 percentage points.
[32] Jones et al., 2022	Rapid Deployment of Team Nursing During a Pandemic: Implementation Strategies and Lessons Learned	*Critical Care Nurse*	Healthcare teams during a pandemic	Descriptive study design. A team nursing model was utilized, and education and resources were developed to distinguish team nurses from intensive care unit nurses, introduce them to the intensive care unit environment, outline expectations, communicate between team nursing pairs, and guide charge nurses in making staffing decisions and assignments.	The pilot program provided positive outcomes that included a reduced need for float nurses and a self-perceived reduction in nursing workload. Rapid deployment of team nursing during a pandemic, implementation lessons identified.
[33] Olaussen et al., 2022	Integrating simulation training during clinical practice in nursing homes: an experimental study of nursing students’ knowledge acquisition, self-efficacy and learning needs	*BMC*	Nursing students in nursing homes	Experimental study integrating simulation training during clinical practice for nursing students. Pre- and post-test comparison studies of simulation training were conducted on nursing students.	The results demonstrate that simulation training improved self-efficacy and was better than other known similar testing methods.
[34] Phillips et al., 2022	The impact of the work environment on the health-related quality of life of Licensed Practical Nurses: a cross-sectional survey in four work environments	*BMC*	Licensed practical nurses in four settings	Cross-sectional analysis. Study reporting how LPNs report their health related to work by using a cross sectional survey administered online.	The study suggests that nurses’ work environment could affect their health. Impact of the work environment on the health-related quality of life was identified as a nurse burnout variable.
[35] Kim et al., 2022	Combination Relationship between Features of Person-Centered Care and Patient Safety Activities of Nurses Working in Small–Medium-Sized Hospitals: A Cross-Sectional Study	*Jiwon Nursing Reports*	Nurses working in small–medium hospitals	Cross-sectional design. Study evaluating 171 nurses from seven small and medium facilities.	The study showed that improved working environment and culture improved patient care. A positive patient safety culture and better nursing environment were both shown to improve patient care and a negative patient safety and culture environment was related to poor communication.
[36] Horvath and Carter, 2022	Emergency Nurses’ Perceptions of Leadership Strategies and Intention to Leave: A scoping review of the literature	*Canadian Journal of Emergency Nursing*	Emergency nurses	This study reviewed how the retention of registered nurses in emergency departments is a critical issue and was further exaggerated by the COVID-19 pandemic.	The study identified that leadership strategies showing support from provider engagement and the organizations influenced nurses’ intention to stay. Emergency nurses’ perceptions of leadership strategies and intention to leave were identified.
[37] Nuruzzaman et al., 2022	Adopting workload-based staffing norms at public sector health facilities in Bangladesh: evidence from two districts	*BMC*	Public-sector health facilities in Bangladesh	This descriptive research discussed the severe shortage and distribution of the healthcare workforce in Bangladesh. Health facilities that were performing well in serving patients during 2016 and 2017 were assessed through examining the staffing requirements of 20 staff categories.	The study identified there was a 50% to 60% increase in the workload of the healthcare workers. The adoption of workload-based staffing norms at public-sector health facilities in Bangladesh was challenged. It concluded that the existing staffing norms fell short of the staffing requirements.
[38] Hasselblad and Loan, 2022	Implementing Shared Governance to Improve Ambulatory Care Nurse Perceptions of Practice Autonomy	*AAACN Viewpoint*	Ambulatory care nurses	Descriptive study. The authors selected and reviewed the results of the 2018 PES-NWI survey from five of the ambulatory care facilities that participated, indicating a poor perception of autonomy by the nurses and a review of the current governance.	The results indicate that after the implementation of improvement strategies, the nurses reported a statistically significant difference in nurses feeling like they have a say in their work environment and the decisions that affect them. Specifically, the implementation of shared governance helped to improve ambulatory care nurse perceptions of autonomy.
[39] HakemZadeh et al., 2022	Differential Relationships Between Work-Life Interface Constructs and Intention to Stay in or Leave the Profession: Evidence from Midwives in Canada	*Psychological Reports*	Midwives in Canada	The descriptive analysis explored how a negative work–life balance is associated with a person’s intentions to stay or leave their profession. An online survey that targeted all the midwives in Canada and was advertised and distributed through the Canadian Association of Midwives and advertised on Facebook, with bookmarks about factors that would influence their decision to stay or leave their profession.	It was identified that if a healthcare organization wants to increase their providers’ intent to stay, their focus should be on reducing the interference of work with the personal lives of their providers. Relationships between work–life interface constructs and intention to stay or leave midwifery were identified.
[40] Unsworth, 2022	In plain sight: the untapped potential of district nurses	*Journal of Community Nursing*	District nurses	Advocated for the expansion of and investment into mainstream services and district nurses rather than investing money into the unknown “newest technology”. Methods used were review of surveys, assessments, and use of knowledge from experience in order to collect data to back up the author’s point of view.	The author found that hospitals and healthcare organizations are spending lots of money on investing in the next big thing, rather than investing in, enhancing, and utilizing the skilled nurses who are available on-site in their district to provide care, which has led to higher openings for nurses and contributed to the nursing shortage.

## Data Availability

Not applicable.
